# Recent progress in LyP-1-based strategies for targeted imaging and therapy

**DOI:** 10.1080/10717544.2019.1587047

**Published:** 2019-03-24

**Authors:** Ningning Song, Lingzhou Zhao, Meilin Zhu, Jinhua Zhao

**Affiliations:** a Department of Nuclear Medicine, Shanghai General Hospital, Shanghai Jiao Tong University School of Medicine, Shanghai, People’s Republic of China;; b School of Basic Medical Sciences, Ningxia Medical University, Yinchuan, People’s Republic of China

**Keywords:** LyP-1, tumor, metastatic lesions, atherosclerotic plaque, diagnosis and therapeutics

## Abstract

The identification of markers expressed by pathological cells or their microenvironment would help to distinguish such cells from the normal tissues. The strategies derived from this theory can be a promising modality for imaging and treating diseases. LyP-1, a tumor homing peptide, can selectively bind to its receptor p32 protein overexpressed in various tumor-associated cells and atherosclerotic plaque macrophages. During recent decades, multiple types of LyP-1-based imaging probes and drug delivery systems have been designed and developed for diagnostic and therapeutic applications. This review first introduces LyP-1 and its receptor p32, as well as its homing, internalization and proapoptotic properties. Next, we highlight recent studies focusing on the applications of LyP-1-based strategies in the diagnosis and treatment of tumors, metastatic lesions, and atherosclerotic plaques. Finally, several limitations in the clinical translation of LyP-1-based bioconjugates are summarized.

## Introduction

Pathological cells and their surrounding microenvironment show a complicated but unique set of molecules that differ from those on the surface of normal cells (Enback & Laakkonen, 2007). For identifying the heterogeneity, phage display techniques have yielded a number of effective and promising small targeting antibodies or homing peptides for the recognition of pathological tissues (David, 2017). Currently, at least 20 antibodies and peptides derived from phage display libraries are under clinical or preclinical studies, and the most successful ones have been approved for clinical use (Omidfar & Daneshpour, 2015). Antibodies or homing peptides-mediated active targeting strategies can deliver payloads such as imaging agents and drugs, to specific diseased tissue and avoid accumulation in nonspecific healthy tissue (Wang et al., 2013; Casi & Neri, 2015; Seidi et al., 2018). Compared to passive targeting, active targeting system is believed to be more efficiency in improving treatment and reducing side effects (Greco & Vicent, 2009; Vlahov & Leamon, 2012).

Unlike antibodies, homing peptides with much smaller molecular size that allow for faster clearance and lower immunogenicity can be of significant value for clinical use (Zhang et al., 2012). Among the existing homing peptides, LyP-1 was identified to recognize tumor cells other than the endothelia of tumor blood vessels (Laakkonen et al., 2002). At first, Laakkonen et al. established a phage display screening using a CX_7_C cyclic peptide library. Then, before initial selection in cell suspensions prepared from MDA-MB-435 human cancer xenografts, anti-CD31 magnetic beads were used for reducing the number of blood vessel endothelial cells, and a phage pool was yielded after three cycles of *ex vivo* selection. Finally, the phage pool was used for *in vivo* selection, and they found that LyP-1-phage could bind to MDA-MB-435 tumor tissue 60 times more efficiently than non-recombinant phage, sugessting LyP-1 was a promising tumor homing peptide. Further experiments showed that LyP-1 could specially target tumor lymphatics, tumor cells and tumor-associated macrophages/myeloid cells (Laakkonen et al., 2002; Enback & Laakkonen, 2007). It is worth noting that the LyP-1-targeted macrophages are not limited to tumor-related cells but also contain activated macrophages in atherosclerotic plaques (Wilson et al., 2009; Uchida et al., 2011). The primary binding site for LyP-1 is a mitochondrial protein named p32, which has been reported to be both overexpressed and aberrantly located at the cell surface of various tumors, especially breast cancer (Fogal et al., 2008). Additionally, due to the fact that p32-positive cells are mainly distributed in hypoxic or nutrient-deprived regions and hypoxia is one of the causes of chemoradiation resistance, treatment strategies based on LyP-1 may be potentially used as a powerful pathway for chemoradiation-resistant cancer treatment (Laakkonen et al., 2004; Fogal et al., 2008). Thus, these unique features of LyP-1 offer an opportunity to explore this molecule as a vector for delivering imaging agents or therapeutic drugs to tumors, metastatic lesions, and atherosclerotic plaques.

To date, LyP-1-incorporated theranostic strategies have been successfully prepared in a variety of forms, including radiolabeled, fluorescent, and nanoparticle-based bioconjugates. Because of their good biocompatibility, nontoxicity, ready modification and controllable size (Lin et al., 2014), nanoparticles (NPs) are preferred in the preparation of such strategy. Moreover, some kinds of NPs are inherently imaging contrast agents and can be a dual-modal imaging agent when co-loaded with other imaging molecules; for example, magnetic iron oxide (Fe_3_O_4_) NPs labeled with a fluorescent dye can be used for magnetic resonance (MR)/fluorescence imaging (Jiang et al., 2017). Another advantage of NPs is the high loading capacity of hydrophilic and lipophilic drugs, which makes them suitable as a therapeutic drug delivery carrier for disease treatment (Lin et al., 2014). Notably, some NPs alone such as bismuth and near-infrared (NIR) absorbing dyes can be applied for local thermotherapy due to their ability to generate heat activated by appropriate external energy sources (Toraya-Brown & Fiering, 2014). Therefore, versatile NPs provide LyP-1-based bioconjugates a platform for building various imaging probes and therapeutic drug delivery systems.

With the current satisfactory efficiency in the imaging and treatment of tumors, metastatic lesions and atherosclerotic plaques, it is necessary to summarize the recent progress in the development of LyP-1-based strategies for diagnosis and therapy of these diseases. In this review, we introduce LyP-1 and its receptor p32, as well as the homing, internalization and proapoptotic properties of LyP-1. Then, the applications of LyP-1-based bioconjugates for diagnostics and therapeutics are discussed, and three other forms of LyP-1 for designing highly stable structures are also reviewed. Finally, we briefly discuss the perspectives and challenges for the clinical application of this technology.

## LyP-1

### LyP-1 and its receptor p32

LyP-1 is a synthetic nonapeptide that was first isolated by phage display screening using MDA-MB-435 human cancer xenografts in 2002 (Laakkonen et al., 2002). The amino acid sequence of LyP-1 is CGNKRTRGC with a molecular weight of 994 Daltons (Timur et al., 2017). The two cysteines in the LyP-1 peptide can be in the form of a disulfide bond and make it a loop (Kotamraju et al., 2015). The linear and cyclic structure of LyP-1 was illustrated in a published study (Timur et al., 2018).

The p32 protein, the cellular receptor of LyP-1, is a doughnut-shaped multicompartmental trimer containing three homologous subunits (A, B and C) (Jiang et al., 1999). Generally, p32 is a mitochondrial matrix protein in normal tissues (Deb & Datta, 1996; Dedio et al., 1998; Matthews & Russell, 1998), but it can also be detected on the cell surface (Soltys et al., 2000), and in the endoplasmic reticulum (Murakami et al., 1993) and nucleus (Matthews & Russell, 1998). Although its functional role in mammalian cells remains elusive, p32 is described a multifunctional chaperone protein, and able to combine with many proteins such as nuclear splicing factor 2, globular domain of C1q and HIV Tat (Storz et al., 2000; Fogal et al., 2008). In addition, several studies have demonstrated that an effect on maintaining oxidative phosphorylation was shown by p32 in yeast (Muta et al., 1997), and autophagy could be induced *via* knocking down the protein level of p32 (Dedio et al., 1998) or disrupting the integrity of p32 (Sengupta et al., 2004).

### The homing, penetration and proapoptotic properties of LyP-1

LyP-1 homing to the desired location is dependent on the recognition of p32 expressed on the cell membrane of certain diseased cells, and the molecular interaction between LyP-1 and p32 has been studied in detail (Timur et al., 2018; Zhang et al., 2018). These studies showed that the interaction of p32 and LyP-1 was dominated by *van der* Waals/hydrophobic and electrostatic energies, and the inner surface of chain B and chain C of p32 is the main binding sites for LyP-1. Moreover, a series of mutation thermodynamic studies have proven that Asn3, Lys4, Arg5 and Arg7 are the necessary amino acid residues in LyP-1, contributing to the special synaptic recognition of p32 (Yenugonda et al., 2017; Timur et al., 2018; Zhang et al., 2018). Formation of the LyP-1 and p32 complex is further demonstrated to be favorable in water due to the negative total binding enthalpy energy (−66.45 ± 7.40 kcal/mol), and the inner surface of p32 is a hanging loop consisting of Phe211-Leu226 in chain A, Glu212-Ser266 in chain B and Glu212-Tyr224 in chain C, which is believed to be helpful in reducing the hydrophobic interactions and facilitating the binding of LyP-1 to p32 (Timur et al., 2018).

**Figure 1.**
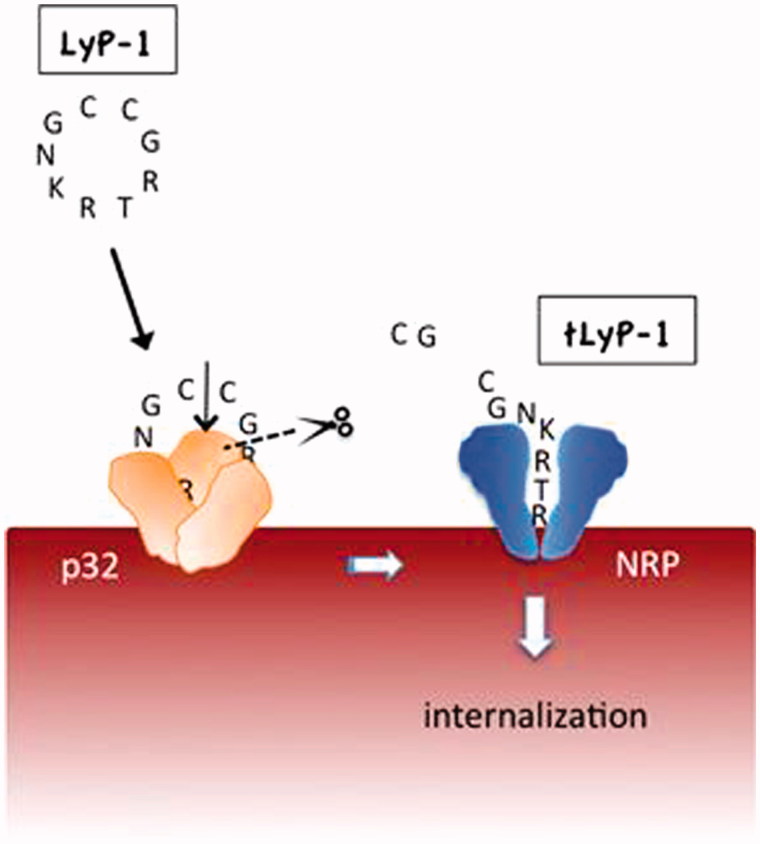
LyP-1 is a cryptic CendR peptide. Cyclic LyP-1 concentrates at the surface of tumor cells by binding to its primary receptor p32. LyP-1 is then proteolytically cleaved into the linear truncated form, tLyP-1, which diminishes its affinity for p32. The exposed C-terminal CendR motif becomes active and triggers binding to NRP1 and/or NRP2, and subsequent cell internalization (adapted from Roth et al., 2012).

LyP-1 not only shows the characteristics of homing to certain pathological cells expressing p32 on their surface, but also can be internalized by these target cells, which is important for the transport of attached cargo into cells. The potential mechanism of this cell-internalizing and tumor-penetrating activity is presumably due to the C-terminal C-end rule (CendR) motif existing on LyP-1 (Sugahara et al., 2009; Teesalu et al., 2009; Sugahara et al., 2010; Roth et al., 2012). CendR peptides are defined by the sequence of (R/K)XX(R/K) (in which X represents any amino acid), which can activate transvascular transport, cell internalization, and parenchymal penetration through a high affinity with neuropilin-1/2 (NRP1/2) on pathological cells. The LyP-1 internalization through CendR pathway is shown Figure 1. When LyP-1 (CGN**KRTR**GC, the bolded residues show the CendR segment) combines with the p32 receptor, and it undergoes proteolytic processing to expose the CendR peptide (CGNKRTR, also called tLyP-1). Subsequently, the CendR peptide is able to bind to NRP1 or NRP2, the second receptor with an ability of triggering the transport pathway for extravasation and tissue penetration (Hamzah et al., 2011). The internalization capacity of LyP-1 gifted by CendR sequence allows LyP-1 and its modified cargoes to enter not only the inside of tumor tissues but also the cell nucleus, which makes it more effective in imaging and treatment of diseases.

In addition, LyP-1 can induce apoptosis in target cells, making it a unique tumor homing peptide (Enback & Laakkonen, 2007). An early study showed that LyP-1 could selectively bind to breast cancer cells and induce cell death *in vitro*, and the proapoptotic activity and the tumor inhibiting effects of LyP-1 were also observed *in vivo* (Laakkonen et al., 2004). Another study revealed a significant level of apoptosis in macrophages treated with LyP-1 when compared with these treated with control peptide in atherosclerotic plaque models (She et al., 2016). It should be noted that the proapoptotic effect of the former was achieved through systemic exposure of LyP-1 to cancer cells, while the latter by enhancing the hypoxia region. Although the above evidence could show LyP-1 has a proapoptotic effect upon to the binding pathological cells, the mechanism by which LyP-1 induces apoptosis remains unclear. Some studies showed that this might involve its receptor p32, a critical regulator of the balance between oxidative phosphorylation and glycolysis (Fogal et al., 2010; Hamzah et al., 2011; She et al., 2016).

### Diagnostic applications for LyP-1-based strategies

The satisfactory targeting and internalization ability of LyP-1 and its potential proapoptotic properties described above endow it an attractive homing peptide for ongoing researches in the fields of disease imaging and therapy (Jiang et al., 1999; Laakkonen et al., 2004; Teesalu et al., 2009; Sugahara et al., 2010). To date, imaging agents such as fluorescent dyes, radionuclides have been directly labeled with LyP-1, and various nanomaterials including but not limited to Fe_3_O_4_ NPs, bismuth (Bi), Abraxane, microbubbles (MBs), liposomes, heat shock protein (Hsp) and dendrimers have been selected as platforms for preparing multifunctional Lyp-1-modified complexes.

## Tumors

The synaptic recognition of LyP-1 with p32 is specific in certain cancers but not in others. It has been proven that the targeted cancer cell lines include MDA-MB-231, MCF7, 4T1, MMTV-PyMT, MDA-MB-435, MDA-MB-435S, B16-F1, Pan02, 22Rv1, SPC-A1, Raji, TRAMP, OVCAR-4, HepG2 and KRIB (Fogal et al., 2008; Laakkonen et al., 2008; Herringson & Altin, 2011; Ren et al., 2012; Yan et al., 2012; Kotamraju et al., 2015; Jiang et al., 2017). Researchers have also detected significantly higher levels of p32 expressed on tumors and stroma in clinical samples of human carcinomas, such as breast cancer, pancreatic cancer, melanoma, endometrioid adenocarcinoma, and colon cancer, than those expressed in corresponding nonmalignant tissues (Fogal et al., 2008; Jiang et al., 2017).

LyP-1 has been labeled with fluorescent dyes for targeting imaging *in vitro* and *in vivo*. To investigate the accumulation of LyP-1 in tumor tissues, fluorescein isothiocyanate isomer (FITC) was modified at the N-terminus of peptides, and the FITC-LyP-1 or FITC-control peptide (ARALPSQRSR) was administrated *via* tail vein to MDA-MB-435 and C8161 xenografts, respectively. The results revealed that strong fluorescence could be observed only in tumors excised from FITC-LyP-1-treated mice bearing MDA-MB-435, but little fluorescence was found in other organs such as liver, and in the mice treated with FITC-control peptide. However, these remarkable results were not observed in mice bearing C8161 tumor cells, suggesting this kind of tumor cell lines do not bind LyP-1. In this study, EF5 was also used as a hypoxia probe to confirm the hypoxic areas, and the accumulated region of LyP-1 was similar to the area where EF5 localized, indicating that LyP-1 targeted tumor areas are tumor microenvironments under hypoxia (Laakkonen et al., 2004). The extravascular accumulation of LyP-1 *in vivo* was further proven by other studies in tumor models bearing 22Rv1 (Kotamraju et al., 2015) or MDA-MB-435 cells (Fogal et al., 2008; von Maltzahn et al., 2008).

Radionuclide imaging is an important tool for diagnosis in the clinic due to its high sensitivity and excellent signal quantification (Bailey & Willowson, 2014). LyP-1 was labeled with ^131^I *via* a tyrosine added at their N-termini for separating the nuclide from sequence. In this study, the tumor area could be clearly detected by single-photon emission computed tomography (SPECT) imaging at 6 h after intravenous injection of ^131^I-LyP-1 in tumor-bearing mice with MDA-MB-435 cells, and the ratios of tumor to muscle and tumor to blood were 6.3 and 1.1, respectively. However, the ^131^I-control peptide (CGGGGGGGC) did not show these advantages (Yu et al., 2013). In another study, researchers prepared [4-^14^C]-cholesterol labeled LyP-1-targeted liposomes containing phosphatidylethanolamine polyethylene glycol_2000_ (PE-PEG_2000_) or PE-PEG_750_ (to evaluate the effect of PE-PEG length on the interaction of liposomes-anchored moieties to receptors) and administrated them to tumor-bearing C57BL/6 mice with B16-F1 cells. The results showed that the greatest accumulation of ^14 ^C occurred in the tumor, spleen, liver and lungs, while liposomes containing PE-PEG_2000_ or PE-PEG_750_ did not show remarkable differences in the biodistribution in tumors or other organs (Herringson & Altin, 2011).

**Figure 2.**
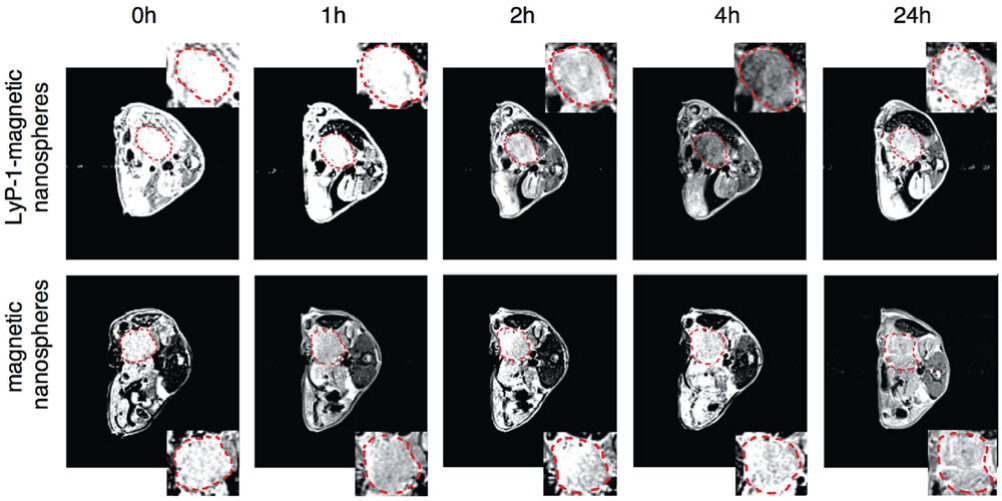
MR scan of orthotopic pancreatic tumor bearing mice at 3T. The T2 weight MR images of orthotopic pancreatic cancer (circled by red dash line) in C57BL/6 mice before and after administration of the Fe_3_O_4_@SiO_2_-FITC@mSiO_2_-LyP-1 or Fe_3_O_4_@SiO_2_-FITC@mSiO_2_ systemically at different time point. The inset is the enlarged pictures of corresponding tumor region (adapted from Jiang et al., 2017).

Fe_3_O_4_ NPs are alternative MR contrast agents because of their ability to remarkably shorten proton relaxation and their strong hypointense T2 signal in the targeted area (Liu et al., 2013). The chemical structure of these Fe_3_O_4_ NPs made them powerful platforms to build various multifunctional nanosystems for conjugation with targeting peptides, fluorescent molecules, nuclides, drugs and other molecules (Wahajuddin & Arora, 2012; Laurent et al., 2014). Two recent studies reported the conjugation of Fe_3_O_4_ NPs with LyP-1 and a fluorescence dye as a dual MR/fluorescent imaging agent (Jiang et al., 2017; Abulrob et al., 2018). One study designed a 50 nm multifunctional superparamagnetic mesoporous nanosphere conjugated with LyP-1, and the nanospheres had a core of silica-protected Fe_3_O_4_ and a shell of FITC-labeled mesoporous silica (Fe_3_O_4_@SiO_2_-FITC@mSiO_2_). After incubation with Pan02 and MS1cells *in vitro*, strong fluorescence was only found in Pan02 cells treated with Fe_3_O_4_@SiO_2_-FITC@mSiO_2_-LyP-1, while no apparent fluorescence in Pan02 cells treated with Fe_3_O_4_@SiO_2_-FITC@mSiO_2_ or in MS1 cells treated with Fe_3_O_4_@SiO_2_-FITC@mSiO_2_ with or without LyP-1. This could be explained by the higher level of p32 expressed on the Pan02 cell membrane, which was confirmed by western blot in this study. The targeting ability *in vivo* was further demonstrated in a Pan02 orthotopic pancreatic tumor model. The results showed that the T2 signal was the lowest at 4 h postinjection and maintained at the tumor site for 24 h in the tumor-bearing mice injected with Fe_3_O_4_@SiO_2_-FITC@mSiO_2_-LyP-1. By contrast, no obvious T2 signal change was showed in the tumor-bearing mice receiving Fe_3_O_4_@SiO_2_-FITC@mSiO_2_ (Figure 2) (Jiang et al., 2017). Similarly, in MDA-MB-231 triple-negative breast cancer, both the low T2 values and high fluorescence intensity at the tumor sites showed that LyP-1-Fe_3_O_4_ NPs could be a satisfactory imaging agent for dual MR/fluorescence imaging (Abulrob et al., 2018). In addition, another research found that the accumulation of LyP-1-magnetic nanoworms (NWs)-Cy5.5 in the tumors was increased after the tumors were heated to 45 °C using free gold nanorods under NIR irradiation in an MDA-MB-435 tumor model. This targeting enhancement effect in tumor sites was due to the increased expression level of p32 induced by heat, which was further tested and verified using another nanoscale imaging probe of LyP-1-liposomes *in vivo* (Park et al., 2010).

Bi has been widely used for diagnostic applications such as computed tomography (CT) (Lusic & Grinstaff, 2013) and photoacoustic (PA) imaging (Yu et al., 2017) due to its large atomic number (*Z* = 83) and high absorption of NIR window laser radiation. Kinsella et al. developed LyP-1-labeled bismuth sulfide (Bi_2_S_3_) NPs as a novel X-ray CT contrast agent (Kinsella et al., 2011). Quantitative analysis showed that the amount of Bi in tumor receiving LyP-1-Bi_2_S_3_ NPs was 1.7-fold higher than that of tumor receiving Bi_2_S_3_ NPs at 4.5 h post injection. In addition, the liver, spleen, and intestines were organs with high accumulation of Bi at 7 days post injection, indicating that the clearance of these NPs was dominated by the hepatobiliary/fecal route. In another study, a LyP-1-labeled Bi nanosystem with a diameter of only 12 nm was prepared (Yu et al., 2017). In the *in vitro* experiment, Bi showed a remarkably higher slope of the Hounsfield unit (HU) value than iohexol that is the most commonly used CT contrast agent in the clinic, suggesting that Bi may be superior to iodinated CT contrast agents. In a 4T1 tumor-bearing mouse model, LyP-1-Bi NPs also showed excellent HU value and PA signal intensity after intratumoral injection, suggesting that LyP-1-labeled Bi NPs could be utilized as highly effective CT or PA imaging contrast agents.

Abraxane is an albumin-bound paclitaxel (PTX) with 130 nm, which is generally used as a nanoscale carrier for conjugation with active targeting molecules to establish imaging probes. In a study by Karmali et al., an additional cysteine was added at the N-terminus of LyP-1 for conjugation with 5(6)-carboxyfluorescein (FAM), and then LyP-1 was coupled with Abraxane through their cysteine sulfhydryl group using a sulfosuccinimidyl 4-[*N*-maleimidomethyl] cyclohexane-1-carboxylate (sulfo-SMCC) cross-linker. After FAM-LyP-1-Abraxane was injected into mice bearing MDA-MB-435, the results showed that the fluorescence intensity in the tumor was four times stronger than that in liver. To investigate whether the payload into tumor tissue *via* LyP-1 was still intact, a mixed micelle composed of 1,2-distearoyl-*sn*-glycero-3-phosphoethanolamine-N-[maleimide(polyethylene glycol)_2000_] (DSPE-PEG_2000_)-LyP-1 and DSPE-PEG_2000_-FAM were prepared. The results showed the same fluorescence localization in tumors administered with mixed micelles as those administrated with FAM-LyP-1-Abraxane, indicating intact payloads into extravascular tumor sites (Karmali et al., 2009).

MBs can be used as an ultrasound contrast agent due to the inherent ability to provide a signal-to-noise ratio when exposed under an acoustic field (Kiessling et al., 2012). MBs are basically composed of an encapsulating shell and an inner gas core. This structure can facilitate drug and gene loading as well as targeting molecules modification (Martin & Dayton, 2013). Zheng group manufactured LyP-1-labeled microbubbles *via* biotin-avidin linkage technology to study the targeting ability of MBs-LyP-1 to MDA-MB-231 tumor cells in a microfluidic system. The influence of the flow velocity distributions of MBs-LyP-1 on its targeting ability was also explored. In this work, MDA-MB-231 tumor cells were cultured within the microchambers in a microfluidic system. After washing of the cells with MBs-LyP-1, the results of the fluorescence image showed many targeted bubbles adhered to MDA-MB-231, and some moving bubbles flowed through the chamber with a long fluorescent trace. However, no MBs adhered to cancer cells when the cells were washed with MBs (Li et al., 2009). In their further work, better binding ability was achieved using lower flow rate distributions of <0.03 cm/s which was close to that in capillaries (Yan et al., 2014). They also synthesized MBs using flow-focusing units (FFUs) on a novel microfluidic device that had two inlets-gas and liquid. Additionally, the researchers used this device to successfully prepare multifunctional monodispersed MBs loaded with 6-coumarin, PTX, LyP-1, Fe_2_O_3_, and CdTe/ZnS quantum dots (QDs) in the shell of MBs (Jiang et al., 2011). However, the applications and efficiencies of these multifunctional monodispersed MBs lacked further research *in vitro* and *in vivo*.

### Metastatic lesions

Due to the inherent advantages in the recognition of tumor cells, tumor lymphatics and tumor-associated macrophages, different LyP-1-based imaging agents have been applied in metastatic tumors in lymph nodes (LNs) and lung. In addition, lymphangiogenesis, a tumor-induced pre-metastatic change in suspected LNs (Sleeman, 2015), was also applied in LyP-1 imaging modality agents.

An early research showed that LyP-1 could recognize metastatic lesions and accumulate in metastatic lesions localized in regional LNs and lungs in a melanoma mice model. In this research, the LyP-1-targeted cells were proved to be tumor cells and lymphatic endothelial cells in and around enlarged lymphatic vessels (Laakkonen et al., 2004). For metastatic lymph node (LN) applications, a lymphatic metastatic tumor model was established using BALB/c nu/nu mice with BxPC-3 pancreatic tumor cells inoculated into their nail pads. Three weeks post inoculation, the popliteal, inguinal, iliac and renal hilus LNs were harvested for confirmation of metastasis using H&E staining and pathological examination. Then, the researchers prepared a lymphatic metastatic imaging probe composed of PEG-poly(lactic-co-glycolic acid) (PLGA) NPs conjugated with LyP-1 and coumarin-6 for optical imaging. As expected, the lymphatic metastatic tumors treated with LyP-1-NPs-coumarin-6 displayed significantly higher fluorescence intensity than those treated with NPs-coumarin-6 (Luo et al., 2010). In their further study, the researchers found that the accumulation of LyP-1 was similar to that in regions with tumor associated lymphatics and macrophages in metastatic LNs (Yan et al., 2011).

**Figure 3.**
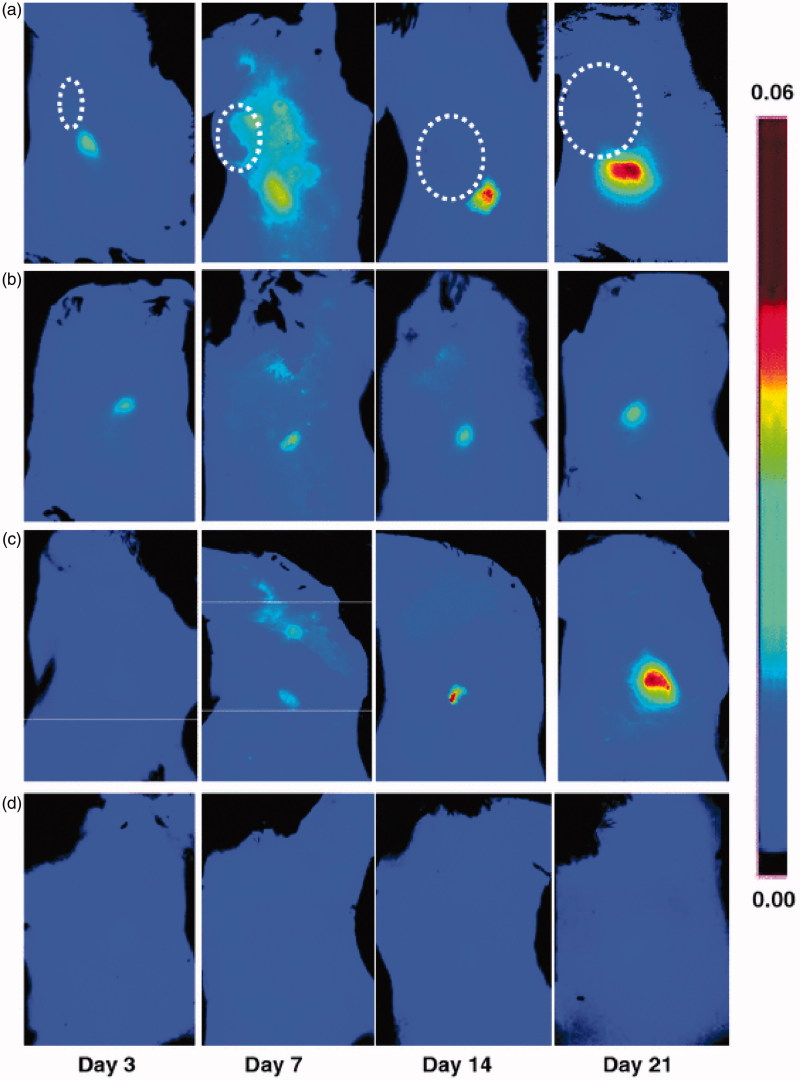
Optical imaging of tumor-induced lymphangiogenesis with Cy5.5-LyP-1. At days 3, 7, 14 and 21 after 4T1 cell inoculation, tumor-bearing BALB/C mice (*n* = 5/group), under isoflurane anesthesia, were injected with Cy5.5-LyP-1 *via* the middle phalanges of both upper extremities. At 45 min after injection, *in vivo* fluorescence imaging of both tumor-draining LNs (a) and contralateral LNs (b) was performed with a Maestro II small-animal *in vivo* imaging system. The fluorescence images consisting of Cy5.5 and autofluorescence spectra were then unmixed based on their spectral patterns with the manufacturer’s software (Maestro software, CRI). The tumors are indicated by *circles*. (c and d) At 24 h post injection, the mice were killed and a dorsal skin flap was elevated to expose the brachial lymph nodes on both sides for a repeat of fluorescence imaging (c. tumor-draining LNs; d. contralateral LNs) (adapted from Zhang et al., 2012).

To evaluate the possibility of imaging lymphangiogenesis in sentinel LNs, LyP-1 was conjugated with a NIR fluorophore at the lysine side chain *via* a reaction with Cy5.5-NHS. The researchers established a collateral brachial LN lymphangiogenesis model by inoculating 4T1 cells into the right shoulders, as primary tumor cells could induce lymphangiogenesis in draining LNs. For *in vivo* optical imaging, the fluorescence intensity of the collateral LNs was found to be significantly higher than that of contralateral LNs at day 7, 14, and 21 after tumor cell inoculation, of which the greatest significance was at day 21. These results were similar to those from *ex vivo* optical imaging. Furthermore, further experiments showed the distribution of LyP-1 was consistent with the lymphangiogenesis region (Figure 3) (Zhang et al., 2012).

### Atherosclerotic plaques

Studies have found that, in addition to being found in certain tumors, p32 was also detected in atherosclerotic lesions with a heavy accumulation in activated macrophages (Peerschke et al., 2004). Since the presence of activated macrophages is believed to play an important role in promoting the initiation, progression and destabilization of atherosclerotic lesions (Libby & Aikawa, 2002; Viola & Soehnlein, 2015), imaging of macrophages could be useful for detecting early-stage atherosclerosis and assessing the risk of plaque rupture (Koenig & Khuseyinova, 2007). Additionally, a high level of p32 has been detected in macrophages in occluded arteries and carotid plaques analyzed in human samples (She et al., 2016). Therefore, tremendous effort has been devoted to developing LyP-1-targeted imaging agents for the diagnosis of atherosclerotic plaques.

**Figure 4.**
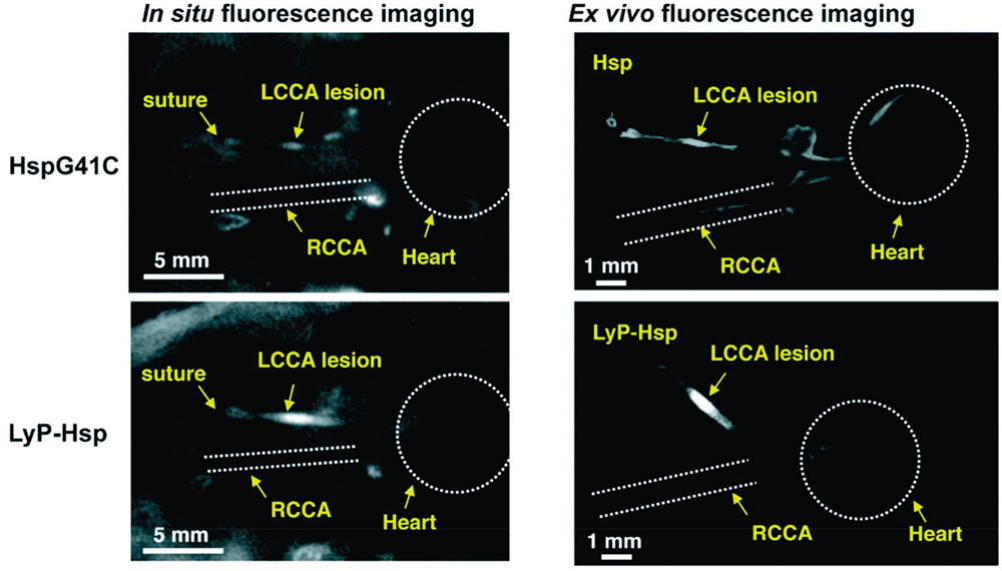
*In situ* and *ex vivo* fluorescence imaging of the mice after 48 h of Cy5.5 labeled HspG41C or LyP-Hsp injection. In both the *in situ* and *ex vivo* images, the LyP-Hsp injected mouse provided more intense fluorescence signal from the left common carotid artery (LCCA) than the right common carotid artery (RCAA) (adapted from Uchida et al., 2011).

Uchida et al. prepared a genetically engineered heat shock protein cage isolated from *Methanococcus jannaschii* (MjHsp) with an exterior diameter of 12 nm and an interior cavity of 9 nm in diameter as a nanoscale carrier. *In vitro*, there was no difference in the geometric mean fluorescence intensity value when LyP-1-MjHsp or MjHsp was incubated with THP-1 monocytes, while the value was significantly higher for LyP-1-MjHsp than MjHsp when THP-1 monoytes were differentiated into macrophages. Next, the researchers established a mouse model of macrophage-rich lesions in left common carotid arteries (LCCAs) to simulate arteriosclerotic plaques. The results showed that the fluorescence signal in the LCCAs was much higher in mice treated with Cy5.5-MjHsp-LyP-1 than those treated with Cy5.5-MjHsp (Figure 4). The location of Cy5.5 was further proven to be positive for Mac-3, a macrophage marker (Uchida et al., 2011). The potential of LyP-1 as a targeting reagent to deliver a superparamagnetic iron oxide NWs-based MR imaging probe and an [^18^F] fluorobenzoic acid-based PET contrast agent to plaques was also confirmed in a published study (Hamzah et al., 2011). Another noteworthy study reported dendrimers as a platform to conjugate with LyP-1 ligands or ARAL peptides (a control peptide, ARALPSQRSR). After radiolabeling of ^64^Cu, the radioactivity of (LyP-1)_4_-dendrimer-^64^Cu accumulated in atherosclerotic plaques of the aorta was remarkably higher than that of (ARAL)_4_-dendrimer-^64^Cu. The ratio of the aorta to blood in mice treated with (LyP-1)_4_-dendrimer-^64^Cu in their study was approximately 3-fold higher than that of the mice treated with 4-[^18^F]fluorobenzoic acid ([^18^F]FBA)-LyP-1, suggesting that the dendrimer was useful in improving the delivery of imaging agents to plaques. Alternatively, the administration route *via* subcutaneous injection was also studied, and the results showed good efficiency, indicating that the dendrimer-based system might be a promising imaging probe in clinical application (Seo et al., 2014).

### Therapeutic applications for LyP-1-based strategies

Conventional chemotherapy and radiotherapy are faced with a challenging goal, which is to enhance local drug concentration and reduce systemic toxic effect. In addressing this challenge, active targeting strategies show promising prospects because they can deliver therapeutic agents to pathological cells, allowing the drugs to accumulate in the diseased tissues while reducing the drug burden in normal tissues. To date, the LyP-1 homing peptide that recognizes the p32 receptor expressed on cell membranes has been widely used to deliver drugs to tumor cells, tumor-associated lymphatic vessels, and macrophages (both in tumors and arteriosclerotic plaques).

In this section, we will discuss various drug delivery systems in the therapeutic efficiency. Drugs applied in these systems included PTX, doxorubicin (DOX), and the antimalarial drug artemisinin (ART). These NPs, such as Bi, IR820 and supermagnetic iron oxide NPs (SPIONs) were used in thermotherapy and evaluated alone or combined with radiotherapy or chemotherapy and photodynamic therapy in cancer. Subsequently, the ability of LyP-1 to deliver genes or siRNAs was also discussed in cancer. In particular, DOX was loaded onto liposomes and evaluated in metastatic LN models. Finally, the capacity of LyP-1 in the proapoptosis effect was applied in atherosclerotic plaques.

## Tumors

PTX, one of the most effective conventional cytotoxic drugs, has been widely used for therapy of different types of tumors (Nehate et al., 2014). The LyP-1 targeted PTX delivery system was investigated by two studies. One of the studies selected MBs as a nanocarrier to evaluate its capacity to deliver PTX into MDA-MB-231 *in vitro*. In this study, MBs were conjugated with LyP-1 and loaded with PTX, and this complex was broken up when exposed to ultrasound. The results showed that, with or without ultrasound irradiation, morphological changes (detached and round appearance) under microscopy were more obvious in LyP-1-labeled PTX-loaded MBs compared to PTX-loaded MBs or unloaded MBs. MTT analysis showed a significantly lower cell viability in LyP-1-labeled PTX-loaded MBs with ultrasound irradiation than that in any of the other protocols, suggesting that LyP-1-labeled MBs could increase the delivery efficiency of PTX (Yan et al., 2011). Abraxane, an albumin-bound PTX, was modified with LyP-1 or CREKA *via* their cysteine sulfhydryl group using a sulfo-SMCC cross-linker to evaluate the inhibition of tumor growth in tumor-bearing mice with MDA-MB-435 cells. After three cycles of a four-times-a-week treatment, LyP-1-Abraxane showed a significantly superior inhibition of tumor growth compared to that of free LyP-1 or untargeted Abraxane. However, CREKA-labeled Abraxane did not cause a highly significant inhibition in tumor volume compared to that of untargeted Abraxane, suggesting a superior efficiency in delivering Abraxane into tumors using LyP-1 as a targeting ligand instead of CREKA (Karmali et al., 2009).

**Figure 5.**
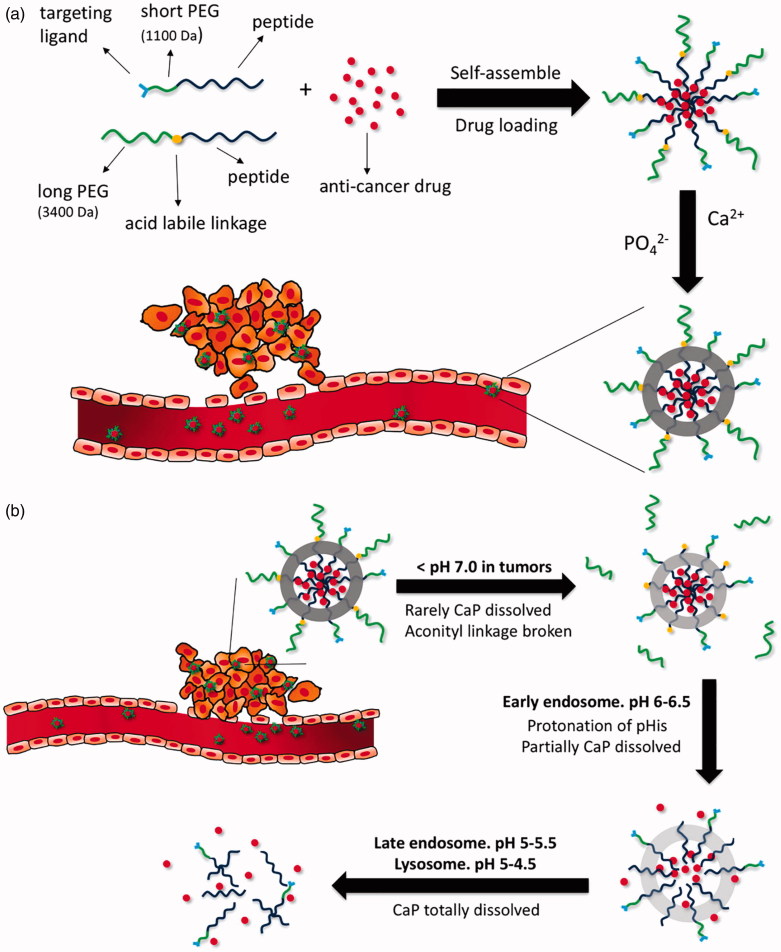
(a) Formation of DOX-loaded nanoparticles with calcium phosphate mineralization layer. (b) Illustrative structural transition of nanoparticles at different *in vivo* conditions (adapted from Wang et al., 2017).

DOX is another cytotoxic drug widely used in many cancer types. Similar to that of other chemotherapy drugs, conventional intravenous or intraperitoneal administration of DOX can also cause potentially toxic effects, particularly cumulative cardiotoxicity (Gabizon et al., 2016). To increase bioavailability and enhance tumor accumulation with minimal side effects, DOX was designed to be encapsulated by LyP-1 targeted nanocarriers. One study was dedicated to preparing an oral DOX delivery nanocarrier system composed of DOX-loaded polysaccharide-lecithin reverse micelles as cores and triglyceride shells on the exterior with various concentrations of RGD and LyP-1 ligands named GLD-PL/TG NPs. The results indicated that the higher concentration of the conjugated LyP-1 rather than RGD could be helpful for the nanosystems to inhibit tumor growth in mice bearing MDA-MB-231 tumors (Su et al., 2017). DOX was also evaluated in a mineralized micelle nanocarrier with PEG_3400_ or PEG_1100_ for extending the circulation time, the anionic shell of poly(L-glutamic acid)_15_ for CaP mineralization, the protonation of poly(L-histidine)_10_ for facilitating DOX release at the target site and the hydrophobic core of poly(L-leucine)_10_ for encapsulation of DOX. The complex exhibited sequential pH-responsivity due to three pH-sensitive components, as showed in Figure 5, which was proven *in vitro* using MDA-MB-231 tumor cells (Wang et al., 2017). Of note, in another study, the treatment efficiency of DOX-loaded LyP-1-liposomes was enhanced when combined with pretreatment of thermotherapy in MDA-MB-435 tumor-bearing mice, as high temperature could enhance the accumulation of LyP-1-liposomes in tumor (Park et al., 2010).

ART is an effective antimalarial drug, but it can also be a potential anticancer agent since it contains an endoperoxide moiety that can react with iron to form free radicals that can kill cancer cells (Lai et al., 2005; Lai et al., 2013). Some studies have demonstrated that the anticancer effect of ART could be observed in the inhibition of angiogenesis, proliferation, and metastasis (Lai et al., 2013). Considering the low water solubility and poor bioavailability of ART, Wang et al. established 30 nm drug-loaded nanoscale systems using micelles as a vehicle with encapsulation of ART and modification with LyP-1 for efficiently transporting ART to diseased tissues. Experiments *in vitro* revealed that the apoptotic rate, S-phase inhibition and cell inhibition effect were all significantly higher after LyP-1-micelle-ART administration than that in micelle-ART administration. Similarly, in an orthotopic breast tumor mice model, LyP-1-micelle-ART showed a remarkably higher inhibitory effect on tumor growth than micelle-ART or free ART (Wang et al., 2012).

Thermotherapy is an effective tumor treatment method through heat with lower toxic effect than conventional chemotherapy and radiotherapy, since cancer cells are particularly more vulnerable at hyperthermic temperatures of 40–45 °C than normal cells are (Mallory et al., 2016). Unlike simple ambient heating methods, complex heating techniques using infrared, microwave, ultrasound and radio frequencies, are promising strategies to induce hyperthermia and avoid overheating normal tissues (Habash et al., 2011). LyP-1-based targeted therapy combined with thermotherapy have been studied and showed excellent results in the inhibition of tumor growth. Photothermal therapy using semimetal Bi NPs irradiated with a 1064 nm NIR laser(Yu et al., 2017) or an NIR absorbing dye of IR820 irradiated with a 808 nm NIR laser (Li et al., 2016) were studied in 4T1 tumor cell-based experiments *in vitro* and *in vivo*. Considering that Bi can be used as a radiosensitizer, the Bi-based nanoscale drug carriers were evaluated for the synergistic effects of radiotherapy and photothermal therapy. For thermotherapy using IR820, as it can induce singlet oxygen, IR820 modified micelles were assessed for triple treatment of photothermal therapy, photodynamic therapy, and chemotherapy when co-loaded with docetaxel (DTX) (Li et al., 2016). A recent study evaluated a novel magnetic induction hyperthermia technique in MCF-7 and CT-26 cells (Teo et al., 2018). Because of the property of heat generation under external alternating current magnetic fields, SPIONs were used as a nanoscale vehicle for magnetic induction hyperthermia. The results displayed tumor growth was significantly reduced in the mice treated with LyP-1-SPIONs when compared with those treated with SPIONs. Furthermore, confocal analysis showed that LyP-1-SPIONs could accumulate in the nucleus, the deadly part of the cell, whereas SPIONs were only found in the cytoplasm.

The targeting and internalization capacity of LyP-1 made it a candidate as an active molecule for cancer gene therapy. Oker-Blom et al. selected the virus *Autographa californica* multiple nucleopolyhedrovirus (*Ac*MNPV), a prototype of the *Baculoviridae* family, as a delivery vehicle for gene therapy due to its ready manipulation, high ability to accept foreign DNA and low toxicity (Makela et al., 2006). *In vitro* studies using MDA-MB-435 and HepG2 cells showed that the binding and transduction efficiency of *Ac*MNPV-LyP-1 were remarkably higher than those of *Ac*MNPV without LyP-1. Block experiments proved that *Ac*MNPV played a role in transduction and was enhanced by LyP-1 (Makela et al., 2008). SiRNA technology in tumor therapy remains challenging due to the lack of specific recognition. Ren et al. established the siRNA carrier composed of tandem LyP-1 and cell-penetrating peptides. The results showed that this complex entered into cells by endocytosis and was sequestered in endosomes with appreciable release of siRNA from endosomes (Ren et al., 2012). In their further study, the complex of LyP-1-cell-penetrating-peptide showed excellent capacity in delivering ID4-specified siRNAs in OVCAR-4 human ovarian tumor-bearing mice (Ren et al., 2012). In another study, the positively-charged nanocomplexes of nine arginines conjugated with LyP-1 were prepared for loading negatively-charged siRNA and endosomal release peptide E9, and the results showed satisfactory knockdown and biological response efficiency (Bjorge et al., 2017).

### Metastatic lesions

LyP-1 can be employed to produce excellent images of suspected LNs before metastasis and lymphatic metastatic tumors, which represents two metastatic states in tumor metastasis into regional LNs. However, the efficiency of LyP-1-based carriers for loading and delivering drugs into targeted lymphatic metastatic tumors needs to be validated.

Popliteal LN metastatic mouse models were established by inoculating MDA-MB-435 melanoma cells (Yan et al., 2011) or SPC-A1 lung cancer cells (Yan et al., 2012) *via* the left hind footpad. Then PEG-DSPE liposomes conjugated with LyP-1 and DOX were prepared for treatment *in vitro* and *in vivo*. In an MDA-MB-435 cells inhibition experiment, the IC50 value of LyP-1-liposomes-DOX was significantly lower than that of liposomes-DOX. *In vivo* using MDA-MB-435 tumor-bearing mice, the lymphatic vessel density of popliteal LNs was remarkably lower after the LyP-1-liposomes-DOX treatment when compared to the liposomes-DOX, suggesting the excellent treatment of destruction of tumor-associated lymphatic vessels. Further experiments showed that the differences was remarkable in the terms of weight and volume ratios of left popliteal LNs to those of right popliteal LNs after LyP-1-liposomes-DOX treatment compared to the corresponding rates after liposomes-DOX treatment. Data also showed satisfactory inhibition effect on metastasis rates after treatment with LyP-1-liposomes-DOX. Similar results were observed in the mouse models of lung cancer LN metastasis. The administration involved injection was *via* the hind footpad, and the toxic damage to the hind footpad showed by H&E staining were abnormalities but edema in the epidermal layer of skin tissues after LyP-1-liposomes-DOX treatment compared to that after treatment with free DOX. These data indicated that LyP-1-labeled liposomes were excellent nanoscale carriers for delivering DOX into lymphatic metastatic tumors, significantly destroying tumor-associated lymphatic vessels, inhibiting LN metastases and decreasing damage to the injection site.

### Atherosclerotic plaques

In atherosclerotic plaques, LyP-1 can target various cell types expressing p32 on their surface, which makes LyP-1 a potential active targeting molecule for delivering therapeutic drugs into diseased tissue. However, there has been no reported study to evaluate a LyP-1-based drug delivery system for the treatment of atherosclerotic plaques, except for one study examining the proapoptotic effect of LyP-1 itself in plaque macrophages (She et al., 2016). The researchers established three atherosclerotic plaque models using high fat diet (HFD), HFD combined with complete or partial ligation of the LCCAs in ApoE null mice. After control peptide or LyP-1 treatment for 8 weeks, a remarkable reduction in plaque formation and plaque occupation rates in the LyP-1-treated group was observed in plaque models established by complete or partial ligation of the carotid artery combined with HFD, other than HFD alone. Further hypoxia probe experiments revealed that hypoxia of carotid artery plaques induced by ligation of the carotid artery combined with HFD was more severe than that induced by HFD alone. TUNEL staining experiments showed a higher apoptotic rate in macrophages released from hypoxic plaques after the treatment of LyP-1when compared to control peptide (ARALPSQRSR), suggesting that macrophages in severe hypoxia plaques were more vulnerable to LyP-1 treatment.

### Theranostic applications of other LyP-1-based strategies

With the presence of disulfide bonds, LyP-1 faces the challenge of maintaining stability in the relatively harsh physiological environment *in vivo*. For example, it was reported that the disulfide linkage is rapidly cleaved in the brain, hindering the application of targeting molecules containing disulfide bonds in glioma or metastatic brain tumors (Bickel et al., 1995). To meet this challenge, great efforts have focused on exploring other structural forms of LyP-1 peptide in order to achieve better biostability *in vivo* for cancer diagnosis and treatment.

As a high affinity of the linear form of LyP-1 for p32 had been previously demonstrated by molecular dynamics simulations, a self-microemulsifying drug delivery system (SMEDDS) conjugated with linear LyP-1 was prepared. The MTT analysis showed significantly higher cytotoxicity of linear LyP-1-SMEDDS than of a control SMEDDS in MDA-MB-231 cells (Gursoy & Cevik, 2014). However, there is no study focusing on the comparison of the linear and cyclic forms of LyP-1 in binding to p32 and biostability in the body. Li et al. prepared a so-called Syp-1 peptide in which the disulfide bonds were replaced by diseleno bonds *via* the replacement of cysteine by selenocysteine. Compared to LyP-1, Syp-1 showed higher serum stability with a similar binding affinity to p32. As a result, Syp-1-liposomes complexes exhibited a significantly higher binding affinity than that of LyP-1-liposomes in *in vitro* and *in vivo* experiments using MDA-MB-435 tumor cells. When DOX was loaded into these complexes, Syp-1-liposomes-DOX exhibited remarkable antitumor efficiency in the inhibition of tumor growth compared with LyP-1-liposomes-DOX (Li et al., 2013). In another study, Zhang et al. prepared a LyP-1 derivative (termed ^L^c(LyP-1)) with better stability and targeting than LyP-1, in which the cysteine at the N-terminal of LyP-1 was replaced by alanine for cycling with amido bond. Then, another stable peptide of ^D^c(LyP-1)(the D-peptide isomer of ^L^c(LyP-1)) was designed to compare their capacity in tumor targeting efficiency. The results showed that, although ^D^c(LyP-1) showed excellent stability in rat serum *in vitro*, its binding affinity to the p32 protein was lower than that of ^L^c(LyP-1). Further targeting experiments based on 4T1 cells showed higher fluorescence signals in the ^L^c(LyP-1)-micelle-DiR groups when compared with the ^D^c(LyP-1)-micelle-DiR groups. When PTX was loaded into micelles, the complex conjugated with ^L^c(LyP-1) exhibited more apoptosis than those conjugated with ^D^c(LyP-1). These results indicated that ^L^c(LyP-1) is superior to ^D^c(LyP-1) in targeting and delivering drugs to tumor cells (Zhang et al., 2018).

## Conclusion

Strong interest has been attracted to the bioimaging and therapeutics of LyP-1 peptide bioconjugates, since LyP-1 was screened by a phage display peptide library. Here, we introduced the properties of LyP-1 in homing, internalization and cytoxicity, and summarized recent studies focusing on LyP-1-based bioconjugates in the applications of tumors, metastatic lesions, and atherosclerotic plaques. In tumors, the successful imaging and therapeutic efficiency of LyP-1-based bioconjugates are dependent on p32 expression on the cell surface of diseased cells. For metastasis applications, LyP-1-based bioconjugates showed more diverse in theranostic applications of lymphangiogenesis before metastasis in suspected LNs, metastatic tumors in LNs and lung. Furthermore, the activated macrophages-targeting capacity of LyP-1 made it a useful probe in imaging of atherosclerotic plaques, and prolonged systemic treatment with LyP-1 alone could induce significant apoptosis and inhibit disease development in advanced hypoxic plaques. Although LyP-1-based bioconjugates have shown strong potential in applications for the imaging and therapy of tumors, metastatic lesions, and atherosclerotic plaques, there is still insufficient evidence for several aspects. First, the mechanism of the proapoptotic effect of LyP-1 in target cells is still unclear, and there are not enough studies focusing on free LyP-1 in treatment applications. So some basic research should take more effort to better understand the proapoptotic effect of this peptide. Second, with an affinity to hypoxic regions rather than to microvascular-rich areas, LyP-1 can be a novel pinpoint platform for ferrying chemo-/radio-sensitizers into chemo-/radio-resistant hypoxic regions, because hypoxic regions are considered to be chemo-/radio-resistant sites in tumors. Based on one report using hypoxic exacerbation to improve the efficiency in imaging and treatment in atherosclerosis plaques, further studies focusing on exploiting the hypoxic targeting capacity of LyP-1 are expected to succeed in tumor applications. Third, the presentation of the relatively instable disulfide bond in LyP-1 makes it difficult to remain stable in a harsh physiological environment. As for the three other forms of LyP-1 derivatives, only data comparing Syp-1 and LyP-1 is shown in the study. For this reason, other forms of LyP-1 derivatives are expected to be designed and explored for addressing this issue. Therefore, further advances in LyP-1-based strategies are expected to address these aspects to close the gap in the translation of experimental data to clinical application.

## References

[CIT0001] Abulrob A, Corluka S, Blasiak B, et al. (2018). LyP-1 conjugated nanoparticles for magnetic resonance imaging of triple negative breast cancer. Mol Imaging Biol 20:428–35. 29101636 10.1007/s11307-017-1140-4

[CIT0002] Bailey DL, Willowson KP (2014). Quantitative SPECT/CT: SPECT joins PET as a quantitative imaging modality. Eur J Nucl Med Mol Imaging 41:17–25. 10.1007/s00259-013-2542-4 24037503

[CIT0003] Bickel U, Kang YS, Pardridge WM (1995). *In vivo* cleavability of a disulfide-based chimeric opioid peptide in rat brain. Bioconjug Chem 6:211–8. 7599264 10.1021/bc00032a009

[CIT0004] Bjorge JD, Pang A, Fujita DJ (2017). Delivery of gene targeting siRNAs to breast cancer cells using a multifunctional peptide complex that promotes both targeted delivery and endosomal release. PLoS One 12:e0180578. 28666009 10.1371/journal.pone.0180578 PMC5493434

[CIT0005] Casi G, Neri D (2015). Antibody-drug conjugates and small molecule-drug conjugates: opportunities and challenges for the development of selective anticancer cytotoxic agents. J Med Chem 58:8751–61. 26079148 10.1021/acs.jmedchem.5b00457

[CIT0006] David A (2017). Peptide ligand-modified nanomedicines for targeting cells at the tumor microenvironment. Adv Drug Deliv Rev 119:120–42. 28506743 10.1016/j.addr.2017.05.006

[CIT0085] Deb TB, Datta K. (1996). Molecular cloning of human fibroblast hyaluronic acid-binding protein confirms its identity with P-32, a protein co-purified with splicing factor SF2. Hyaluronic acid-binding protein as P-32 protein, co-purified with splicing factor SF2. J Biol Chem 271:2206–12. 10.1074/jbc.271.4.2206 8567680

[CIT0086] Dedio J, Jahnen-Dechent W, Bachmann M, et al. (1998) The multiligand-binding protein gC1qR, putative C1q receptor, is a mitochondrial protein. J Immunol 160:3534–42. 9531316

[CIT0007] Enback J, Laakkonen P (2007). Tumour-homing peptides: tools for targeting, imaging and destruction. Biochem Soc Trans 35:780–3. 17635147 10.1042/BST0350780

[CIT0008] Fogal V, Richardson AD, Karmali PP, et al. (2010). Mitochondrial p32 protein is a critical regulator of tumor metabolism *via* maintenance of oxidative phosphorylation. Mol Cell Biol 30:1303–18. 20100866 10.1128/MCB.01101-09 PMC2832503

[CIT0009] Fogal V, Zhang L, Krajewski S, et al. (2008). Mitochondrial/cell-surface protein p32/gC1qR as a molecular target in tumor cells and tumor stroma. Cancer Res 68:7210–8. 18757437 10.1158/0008-5472.CAN-07-6752 PMC2562323

[CIT0010] Gabizon AA, Patil Y, La-Beck NM (2016). New insights and evolving role of pegylated liposomal doxorubicin in cancer therapy. Drug Resist Updat 29:90–106. 27912846 10.1016/j.drup.2016.10.003

[CIT0011] Greco F, Vicent MJ (2009). Combination therapy: opportunities and challenges for polymer-drug conjugates as anticancer nanomedicines. Adv Drug Deliv Rev 61:1203–13. 19699247 10.1016/j.addr.2009.05.006

[CIT0012] Gursoy RN, Cevik O (2014). Design, characterization and *in vitro* evaluation of SMEDDS containing an anticancer peptide, linear LyP-1. Pharm Dev Technol 19:486–90. 23678858 10.3109/10837450.2013.795170

[CIT0013] Habash RW, Krewski D, Bansal R, et al. (2011). Principles, applications, risks and benefits of therapeutic hyperthermia. Front Biosci (Elite Ed) 3:1169–81. 21622123 10.2741/e320

[CIT0014] Hamzah J, Kotamraju VR, Seo JW, et al. (2011). Specific penetration and accumulation of a homing peptide within atherosclerotic plaques of apolipoprotein E-deficient mice. Proc Natl Acad Sci USA 108:7154–9. 21482787 10.1073/pnas.1104540108 PMC3084060

[CIT0015] Herringson TP, Altin JG (2011). Effective tumor targeting and enhanced anti-tumor effect of liposomes engrafted with peptides specific for tumor lymphatics and vasculature. Int J Pharm 411:206–14. 21443937 10.1016/j.ijpharm.2011.03.044

[CIT0016] Jiang C, Li X, Yan F, et al. (2011). Microfluidic-assisted formation of multifunctional monodisperse microbubbles for diagnostics and therapeutics. Micro Nano Lett 6:417–21.

[CIT0017] Jiang J, Zhang Y, Krainer AR, et al. (1999). Crystal structure of human p32, a doughnut-shaped acidic mitochondrial matrix protein. Proc Natl Acad Sci USA 96:3572–7. 10097078 10.1073/pnas.96.7.3572 PMC22335

[CIT0018] Jiang Y, Liu S, Zhang Y, et al. (2017). Magnetic mesoporous nanospheres anchored with LyP-1 as an efficient pancreatic cancer probe. Biomaterials 115:9–18. 27871003 10.1016/j.biomaterials.2016.11.006

[CIT0019] Karmali PP, Kotamraju VR, Kastantin M, et al. (2009). Targeting of albumin-embedded paclitaxel nanoparticles to tumors. Nanomedicine 5:73–82. 18829396 10.1016/j.nano.2008.07.007 PMC2824435

[CIT0020] Kiessling F, Fokong S, Koczera P, et al. (2012). Ultrasound microbubbles for molecular diagnosis, therapy, and theranostics. J Nucl Med 53:345–8. 22393225 10.2967/jnumed.111.099754

[CIT0021] Kinsella JM, Jimenez RE, Karmali PP, et al. (2011). X-ray computed omography imaging of breast cancer by using targeted peptide-labeled bismuth sulfide nanoparticles. Angew Chem Int Ed Engl 50:12308–11. 22028313 10.1002/anie.201104507 PMC3530424

[CIT0022] Koenig W, Khuseyinova N (2007). Biomarkers of atherosclerotic plaque instability and rupture. Arterioscler Thromb Vasc Biol 27:15–26. 17082488 10.1161/01.ATV.0000251503.35795.4f

[CIT0023] Kotamraju VR, Sharma S, Kolhar P, et al. (2015). Increasing tumor accessibility with conjugatable disulfide-bridged tumor-penetrating peptides for cancer diagnosis and treatment. Breast Cancer (Auckl) 9:79–87. 27385913 10.4137/BCBCR.S29426 PMC4924884

[CIT0024] Laakkonen P, Akerman ME, Biliran H, et al. (2004). Antitumor activity of a homing peptide that targets tumor lymphatics and tumor cells. Proc Natl Acad Sci USA 101:9381–6. 15197262 10.1073/pnas.0403317101 PMC438985

[CIT0025] Laakkonen P, Porkka K, Hoffman JA, et al. (2002). A tumor-homing peptide with a targeting specificity related to lymphatic vessels. Nat Med 8:751–5. 12053175 10.1038/nm720

[CIT0026] Laakkonen P, Zhang L, Ruoslahti E (2008). Peptide targeting of tumor lymph vessels. Ann NY Acad Sci 1131:37–43. 18519957 10.1196/annals.1413.003

[CIT0027] Lai H, Sasaki T, Singh NP (2005). Targeted treatment of cancer with artemisinin and artemisinin-tagged iron-carrying compounds. Expert Opin Ther Targets 9:995–1007. 16185154 10.1517/14728222.9.5.995

[CIT0028] Lai HC, Singh NP, Sasaki T (2013). Development of artemisinin compounds for cancer treatment. Invest New Drugs 31:230–46. 22935909 10.1007/s10637-012-9873-z

[CIT0029] Laurent S, Saei AA, Behzadi S, et al. (2014). Superparamagnetic iron oxide nanoparticles for delivery of therapeutic agents: opportunities and challenges. Expert Opin Drug Deliv 11:1449–70. 24870351 10.1517/17425247.2014.924501

[CIT0030] Li C, Wang Y, Zhang X, et al. (2013). Tumor-targeted liposomal drug delivery mediated by a diseleno bond-stabilized cyclic peptide. Int J Nanomedicine 8:1051–62. 23515368 10.2147/IJN.S40498 PMC3598503

[CIT0031] Li W, Peng J, Tan L, et al. (2016). Mild photothermal therapy/photodynamic therapy/chemotherapy of breast cancer by Lyp-1 modified Docetaxel/IR820 Co-loaded micelles. Biomaterials 106:119–33. 27561883 10.1016/j.biomaterials.2016.08.016

[CIT0032] Li X, Jin Q, Chen T, et al. (2009). LyP-1 ultrasonic microbubbles targeting to cancer cell as tumor bio-acoustics markers or drug carriers: targeting efficiency evaluation in, microfluidic channels. Conf Proc IEEE Eng Med Biol Soc 2009:463–6. 19964739 10.1109/IEMBS.2009.5334473

[CIT0033] Libby P, Aikawa M (2002). Stabilization of atherosclerotic plaques: new mechanisms and clinical targets. Nat Med 8:1257–62. 12411953 10.1038/nm1102-1257

[CIT0034] Lin PC, Lin S, Wang PC, et al. (2014). Techniques for physicochemical characterization of nanomaterials. Biotechnol Adv 32:711–26. 24252561 10.1016/j.biotechadv.2013.11.006 PMC4024087

[CIT0035] Liu G, Gao J, Ai H, et al. (2013). Applications and potential toxicity of magnetic iron oxide nanoparticles. Small 9:1533–45. 23019129 10.1002/smll.201201531

[CIT0036] Luo G, Yu X, Jin C, et al. (2010). LyP-1-conjugated nanoparticles for targeting drug delivery to lymphatic metastatic tumors. Int J Pharm 385:150–6. 19825404 10.1016/j.ijpharm.2009.10.014

[CIT0037] Lusic H, Grinstaff MW (2013). X-ray-computed tomography contrast agents. Chem Rev 113:1641–66. 23210836 10.1021/cr200358s PMC3878741

[CIT0038] Makela AR, Enback J, Laakkonen JP, et al. (2008). Tumor targeting of baculovirus displaying a lymphatic homing peptide. J Gene Med 10:1019–31. 18655234 10.1002/jgm.1222

[CIT0039] Makela AR, Matilainen H, White DJ, et al. (2006). Enhanced baculovirus-mediated transduction of human cancer cells by tumor-homing peptides. J Virol 80:6603–11. 16775347 10.1128/JVI.00528-06 PMC1488948

[CIT0040] Mallory M, Gogineni E, Jones GC, et al. (2016). Therapeutic hyperthermia: the old, the new, and the upcoming. Crit Rev Oncol Hematol 97:56–64. 26315383 10.1016/j.critrevonc.2015.08.003

[CIT0041] Martin KH, Dayton PA (2013). Current status and prospects for microbubbles in ultrasound theranostics. Wiley Interdiscip Rev Nanomed Nanobiotechnol 5:329–45. 23504911 10.1002/wnan.1219 PMC3822900

[CIT0042] Matthews DA, Russell WC (1998). Adenovirus core protein V interacts with p32–a protein which is associated with both the mitochondria and the nucleus. J Gen Virol 79:1677–85. 9680131 10.1099/0022-1317-79-7-1677

[CIT0043] Murakami H, Blobel G, Pain D (1993). Signal sequence region of mitochondrial precursor proteins binds to mitochondrial import receptor. Proc Natl Acad Sci USA 90:3358–62. 8475080 10.1073/pnas.90.8.3358 PMC46299

[CIT0044] Muta T, Kang D, Kitajima S, et al. (1997). p32 protein, a splicing factor 2-associated protein, is localized in mitochondrial matrix and is functionally important in maintaining oxidative phosphorylation. J Biol Chem 272:24363–70. 9305894 10.1074/jbc.272.39.24363

[CIT0045] Nehate C, Jain S, Saneja A, et al. (2014). Paclitaxel formulations: challenges and novel delivery options. Curr Drug Deliv 11:666–86. 24909147 10.2174/1567201811666140609154949

[CIT0046] Omidfar K, Daneshpour M (2015). Advances in phage display technology for drug discovery. Expert Opin Drug Discov 10:651–69. 25910798 10.1517/17460441.2015.1037738

[CIT0047] Park JH, von Maltzahn G, Xu MJ, et al. (2010). Cooperative nanomaterial system to sensitize, target, and treat tumors. Proc Natl Acad Sci USA 107:981–6. 20080556 10.1073/pnas.0909565107 PMC2824295

[CIT0048] Peerschke EI, Minta JO, Zhou SZ, et al. (2004). Expression of gC1q-R/p33 and its major ligands in human atherosclerotic lesions. Mol Immunol 41:759–66. 15234555 10.1016/j.molimm.2004.04.020

[CIT0049] Ren Y, Cheung HW, von Maltzhan G, et al. (2012). Targeted tumor-penetrating siRNA nanocomplexes for credentialing the ovarian cancer oncogene ID4. Sci Transl Med 4:147ra12. 10.1126/scitranslmed.3003778 PMC3633234 22896676

[CIT0050] Ren Y, Hauert S, Lo JH, et al. (2012). Identification and characterization of receptor-specific peptides for siRNA delivery. ACS Nano 6:8620–31. 22909216 10.1021/nn301975s PMC3478735

[CIT0051] Roth L, Agemy L, Kotamraju VR, et al. (2012). Transtumoral targeting enabled by a novel neuropilin-binding peptide. Oncogene 31:3754–63. 22179825 10.1038/onc.2011.537

[CIT0052] Seidi K, Neubauer HA, Moriggl R, et al. (2018). Tumor target amplification: implications for nano drug delivery systems. J Control Release 275:142–61. 29454742 10.1016/j.jconrel.2018.02.020

[CIT0053] Sengupta A, Tyagi RK, Datta K (2004). Truncated variants of hyaluronan-binding protein 1 bind hyaluronan and induce identical morphological aberrations in COS-1 cells. Biochem J 380:837–44. 15005653 10.1042/BJ20040264 PMC1224209

[CIT0054] Seo JW, Baek H, Mahakian LM, et al. (2014). ^64^Cu-labeled LyP-1-dendrimer for PET-CT imaging of atherosclerotic plaque. Bioconjug Chem 25:231–9. 24433095 10.1021/bc400347s PMC4311647

[CIT0055] She ZG, Hamzah J, Kotamraju VR, et al. (2016). Plaque-penetrating peptide inhibits development of hypoxic atherosclerotic plaque. J Control Release 238:212–20. 27423327 10.1016/j.jconrel.2016.07.020

[CIT0056] Sleeman JP (2015). The lymph node pre-metastatic niche. J Mol Med 93:1173–84. 26489604 10.1007/s00109-015-1351-6

[CIT0057] Soltys BJ, Kang D, Gupta RS (2000). Localization of P32 protein (gC1q-R) in mitochondria and at specific extramitochondrial locations in normal tissues. Histochem Cell Biol 114:245–55. 11083468 10.1007/s004180000191

[CIT0058] Storz P, Hausser A, Link G, et al. (2000). Protein kinase C [micro] is regulated by the multifunctional chaperon protein p32. J Biol Chem 275:24601–7. 10831594 10.1074/jbc.M002964200

[CIT0059] Su CW, Yen CS, Chiang CS, et al. (2017). Multistage continuous targeting with quantitatively controlled peptides on chitosan-lipid nanoparticles with multicore-shell nanoarchitecture for enhanced orally administrated anticancer *in vitro* and *in vivo*. Macromol Biosci 17:1600260. 10.1002/mabi.201600260 27634372

[CIT0060] Sugahara KN, Teesalu T, Karmali PP, et al. (2009). Tissue-penetrating delivery of compounds and nanoparticles into tumors. Cancer Cell 16:510–20. 19962669 10.1016/j.ccr.2009.10.013 PMC2791543

[CIT0061] Sugahara KN, Teesalu T, Karmali PP, et al. (2010). Coadministration of a tumor-penetrating peptide enhances the efficacy of cancer drugs. Science 328:1031–5. 20378772 10.1126/science.1183057 PMC2881692

[CIT0062] Teesalu T, Sugahara KN, Kotamraju VR, et al. (2009). C-end rule peptides mediate neuropilin-1-dependent cell, vascular, and tissue penetration. Proc Natl Acad Sci USA 106:16157–62. 19805273 10.1073/pnas.0908201106 PMC2752543

[CIT0063] Teo P, Wang X, Zhang J, et al. (2018). LyP-1-conjugated Fe_3_O_4_ nanoparticles suppress tumor growth by magnetic induction hyperthermia. J Biomater Sci Polym Ed 29:181–94. 29165044 10.1080/09205063.2017.1409048

[CIT0064] Timur SS, Bhattarai P, Gursoy RN, et al. (2017). Design and *in vitro* evaluation of bispecific complexes and drug conjugates of anticancer peptide, LyP-1 in human breast cancer. Pharm Res 34:352–64. 27896591 10.1007/s11095-016-2066-2

[CIT0065] Timur SS, Yalcin G, Cevik O, et al. (2018). Molecular dynamics, thermodynamic, and mutational binding studies for tumor-specific LyP-1 in complex with p32. J Biomol Struct Dyn 36:1134–44. 28427307 10.1080/07391102.2017.1313779

[CIT0066] Toraya-Brown S, Fiering S (2014). Local tumour hyperthermia as immunotherapy for metastatic cancer. Int J Hyperthermia 30:531–9. 25430985 10.3109/02656736.2014.968640 PMC4558619

[CIT0067] Uchida M, Kosuge H, Terashima M, et al. (2011). Protein cage nanoparticles bearing the LyP-1 peptide for enhanced imaging of macrophage-rich vascular lesions. ACS Nano 5:2493–502. 21391720 10.1021/nn102863y PMC3082619

[CIT0068] Viola J, Soehnlein O (2015). Atherosclerosis - A matter of unresolved inflammation. Semin Immunol 27:184–93. 25865626 10.1016/j.smim.2015.03.013

[CIT0069] Vlahov IR, Leamon CP (2012). Engineering folate-drug conjugates to target cancer: from chemistry to clinic. Bioconjug Chem 23:1357–69. 22667324 10.1021/bc2005522

[CIT0070] von Maltzahn G, Ren Y, Park JH, et al. (2008). *In vivo* tumor cell targeting with "click" nanoparticles. Bioconjug Chem 19:1570–8. 18611045 10.1021/bc800077y PMC2538627

[CIT0071] Wahajuddin, Arora S (2012). Superparamagnetic iron oxide nanoparticles: magnetic nanoplatforms as drug carriers. Int J Nanomedicine 7:3445–71. 22848170 10.2147/IJN.S30320 PMC3405876

[CIT0072] Wang S, Placzek WJ, Stebbins JL, et al. (2013). Novel targeted system to deliver chemotherapeutic drugs to EphA2-expressing cancer cells. J Med Chem 55:2427–36. 10.1021/jm201743s PMC3299084 22329578

[CIT0073] Wang TW, Yeh CW, Kuan CH, et al. (2017). Tailored design of multifunctional and programmable pH-responsive self-assembling polypeptides as drug delivery nanocarrier for cancer therapy. Acta Biomater 58:54–66. 28606810 10.1016/j.actbio.2017.06.008

[CIT0074] Wang Z, Yu Y, Ma J, et al. (2012). LyP-1 modification to enhance delivery of artemisinin or fluorescent probe loaded polymeric micelles to highly metastatic tumor and its lymphatics. Mol Pharm 9:2646–57. 22853186 10.1021/mp3002107

[CIT0075] Wilson HM, Barker RN, Erwig LP (2009). Macrophages: promising targets for the treatment of atherosclerosis. Curr Vasc Pharmacol 7:234–43. 19356007 10.2174/157016109787455635

[CIT0076] Yan F, Li X, Jiang C, et al. (2014). A novel microfluidic chip for assessing dynamic adhesion behavior of cell-targeting microbubbles. Ultrasound Med Biol 40:148–57. 24210864 10.1016/j.ultrasmedbio.2013.09.001

[CIT0077] Yan F, Li X, Jin Q, et al. (2011). Therapeutic ultrasonic microbubbles carrying paclitaxel and LyP-1 peptide: preparation, characterization and application to ultrasound-assisted chemotherapy in breast cancer cells. Ultrasound Med Biol 37:768–79. 21458148 10.1016/j.ultrasmedbio.2011.02.006

[CIT0078] Yan Z, Wang F, Wen Z, et al. (2012). LyP-1-conjugated PEGylated liposomes: a carrier system for targeted therapy of lymphatic metastatic tumor. J Control Release 157:118–25. 21827801 10.1016/j.jconrel.2011.07.034

[CIT0079] Yan Z, Zhan C, Wen Z, et al. (2011). LyP-1-conjugated doxorubicin-loaded liposomes suppress lymphatic metastasis by inhibiting lymph node metastases and destroying tumor lymphatics. Nanotechnology 22:415103. 21914940 10.1088/0957-4484/22/41/415103

[CIT0080] Yenugonda V, Nomura N, Kouznetsova V, et al. (2017). A novel small molecule inhibitor of p32 mitochondrial protein overexpressed in glioma. J Transl Med 15:210. 29047383 10.1186/s12967-017-1312-7 PMC5648515

[CIT0081] Yu MM, Wang RF, Chen YH, et al. (2013). Radiolabeling LyP-1 peptide and preliminary biodistribution evaluation in mice bearing MDA-MB-435 xenografts. Chin Med J 126:471–5. 23422109

[CIT0082] Yu X, Li A, Zhao C, et al. (2017). Ultrasmall semimetal nanoparticles of bismuth for dual-modal computed tomography/photoacoustic imaging and synergistic thermoradiotherapy. ACS Nano 11:3990–4001. 28395135 10.1021/acsnano.7b00476

[CIT0083] Zhang F, Niu G, Lin X, et al. (2012). Imaging tumor-induced sentinel lymph node lymphangiogenesis with LyP-1 peptide. Amino Acids 42:2343–51. 21769497 10.1007/s00726-011-0976-1 PMC3257379

[CIT0084] Zhang X, Wang F, Shen Q, et al. (2018). Structure reconstruction of LyP-1: ^L^c(LyP-1) coupling by amide bond inspires the brain metastatic tumor targeted drug delivery. Mol Pharm 15:430–6. 29215294 10.1021/acs.molpharmaceut.7b00801

